# Is it possible for knowledge-based planning to improve intensity modulated radiation therapy plan quality for planners with different planning experiences in left-sided breast cancer patients?

**DOI:** 10.1186/s13014-017-0822-z

**Published:** 2017-05-22

**Authors:** Juanqi Wang, Weigang Hu, Zhaozhi Yang, Xiaohui Chen, Zhiqiang Wu, Xiaoli Yu, Xiaomao Guo, Saiquan Lu, Kaixuan Li, Gongyi Yu

**Affiliations:** 1 Department of Radiation Oncology, Fudan University Shanghai Cancer Center, Shanghai, China; 2 Department of Oncology, Shanghai Medical College, Fudan University, Shanghai, China

**Keywords:** Intensity modulated radiation therapy, Knowledge-based planning, Breast cancer, Simultaneously integrated boost

## Abstract

**Background:**

Knowledge-based planning (KBP) is a promising technique that can improve plan quality and increase planning efficiency. However, no attempts have been made to extend the domain of KBP for planners with different planning experiences so far. The purpose of this study was to quantify the potential gains for planners with different planning experiences after implementing KBP in intensity modulated radiation therapy (IMRT) plans for left-sided breast cancer patients.

**Methods:**

The model libraries were populated with 80 expert clinical plans from treated patients who previously received left-sided breast-conserving surgery and IMRT with simultaneously integrated boost. The libraries were created on the RapidPlan^TM^. 6 planners with different planning experiences (2 beginner planners, 2 junior planners and 2 senior planners) generated manual and KBP optimized plans for additional 10 patients, similar to those included in the model libraries. The plan qualities were compared between manual and KBP plans.

**Results:**

All plans were capable of achieving the prescription requirement. There were almost no statistically significant differences in terms of the planning target volume (PTV) coverage and dose conformality. It was demonstrated that the doses for most of organs-at-risk (OARs) were on average lower or equal in KBP plans compared to manual plans except for the senior planners, where the very small differences were not statistically significant. KBP data showed a systematic trend to have superior dose sparing at most parameters for the heart and ipsilateral lung. The observed decrease in the doses to these OARs could be achieved, particularly for the beginner and junior planners. Many differences were statistically significant.

**Conclusions:**

It is feasible to generate acceptable IMRT plans after implementing KBP for left-sided breast cancer. KBP helps to effectively improve the quality of IMRT plans against the benchmark of manual plans for less experienced planners without any manual intervention. KBP showed promise for homogenizing the plan quality by transferring planning expertise from more experienced to less experienced planners.

## Background

Intensity modulated radiation therapy (IMRT) has the capability of maintaining the adequate planning target volume (PTV) coverage, without compromising the surrounding critical organs-at-risk (OARs) sparing. In principle, the greatest difficulty in IMRT planning is the determination of the ideal dose–volume constraints for OARs. The most common dose-volume constraints have typically been determined using the Radiation Therapy Oncology Group protocol recommendations, or a physician’s intuition based recommendations. These population-based recommendations can be meaningful in many situations. However, the complex geometric variations between PTV and OARs lead to a large patient-to-patient variation in the OARs sparing. A final treatment plan that meets the guidelines and achieves the clinical acceptance criteria may still be suboptimal for a specific patient, particularly when inexperienced planners are involved. The potential of IMRT can not be fully explored until ideal IMRT optimization algorithms are widely used.

Knowledge-based planning (KBP) is a promising technique that has been demonstrated to improve plan quality and increase planning efficiency [1–12]. This technique is not intended to generate an optimal plan, which depends on other physical and clinical constrains. Rather, it aims to achieve the best plan according to the knowledge of prior plans. RapidPlan™ (Varian Medical Systems, Palo Alto, CA, USA) is a commercially KBP solution that can provide the feasibility of assessing the adequacy of OARs based on the data of clinical treated patients. The RapidPlan™ model libraries quantitatively correlate the information about anatomical geometries and dosimetry. Although the benefits of RapidPlan™ are still being investigated, desirable results have been reported for a range of disease sites and with varying levels of patient complexity using advanced technologies such as IMRT and volumetric modulated arc therapy (VMAT) [13–21]. In these studies, the primary focus was the appraisal of the quality, consistency and efficiency with the help of KBP. However, no attempts have been made to explore the domain of KBP for planners with different planning experiences. The IMRT planning process is demanding [22], and expertise is required to master the various steps in the procedure such as adjusting constraints and priorities to PTVs and OARs. Still, appreciable variation exists in the optimization process, depending on the experience of individual planners. For example, the commonly used simultaneous integrated boost (SIB) treatment scheme for left-sided breast cancer IMRT is quite challenging due to the complexity of the tumor bed shape, dose levels and location. These plans are especially difficult for inexperienced planners, but relatively easy for experienced planners. There is a doubt whether the effect of implementing KBP is consistent for planners with different planning experiences in varying extent. In this study, we mainly aimed to quantify the potential gains for planners with different planning experiences after implementing KBP in IMRT plans for left-sided breast cancer patients.

## Methods

RapidPlan™, which was introduced in the Eclipse treatment planning system (TPS) from the release 13.5, is a novel knowledge-based optimization engine. It allows planners to use the dose and patient anatomy information from treated plans, in the form of dose–volume histogram (DVH) estimation model libraries, to estimate the likely amount of individual OARs sparing that can be achieved in new plans.

### Patient selection and contouring

The patients who previously received left-sided breast-conserving surgery and IMRT with SIB were selected for this study because breast cancer is the major cancer at our institution. The postoperative tumor bed was delineated based on the clips and seroma combined with other postoperative changes. The boost clinical target volume was defined by uniformly adding a margin of 10mm around the tumor bed. An additional margin of 5mm was added to define the boost PTV (PTV_boost_). The breast clinical target volume included the glandular breast tissue of the ipsilateral breast and did not extend into the pectoralis major or the ribs. The breast PTV (PTV_breast_) was defined by adding a margin of 5mm at the anterior-posterior direction and 10mm at the medial-lateral and superior-inferior direction. Both PTV_breast_ and PTV_boost_ were limited superficially 5mm under the skin surface. For optimization and analysis purposes, an elective PTV (PTV_elective_) was constructed, equal to PTV_breast_ minus PTV_boost_ and a transition structure of 4mm between PTV_boost_ and PTV_breast_. The OARs surrounding the PTVs, including the heart, bilateral lungs, and contralateral breast were contoured as well. The heart was contoured from the level of pulmonary trunk superiorly to the apex, including the pericardium but not the major vessels.

### Model configuration

The model libraries were populated with 80 expert clinical plans from treated patients between January 2012 and September 2015. These patients represented a range of breast sizes and body habitus. All plans transferred to RapidPlan™ were originally generated with a commercial TPS (Pinnacle^3^, version 8.0m, Philips Medical Systems, Fitchburg, WI) using the direct machine parameter optimization algorithm, and for the linear accelerators using 6MV photons equipped with millennium 120-leaf multileaf collimators. Each of the plans containing the geometric and dosimetric information in the model libraries was approved by physicians at our institution. The plans aimed to deliver 45Gy to 95% of PTV_breast_ and 60Gy to 95% of PTV_boost_ in 25 fractions, while limiting the volume of each PTV receiving greater than 110% of the prescribed dose. The dose constraints which were derived from our clinical experience and the published studies, are listed as follows: the percent of heart that at least received 5Gy was less than 40% (V_5_≼40%), V_20_≼10%, V_30_≼5% and the mean dose to heart≼6Gy (D_mean_≼6Gy); ipsilateral lung V_5_≼50%,V_20_≼20%, V_30_≼5% and D_mean_≼20Gy; contralateral breast D_mean_≼1Gy and contralateral lung D_mean_≼1Gy. The doses to critical OARs were not further reduced if PTV maximum dose exceeded 110% of the prescription dose, after normalizing to cover 95% of the PTV volume with the prescription dose.

### Evaluation of KBP performance

The evaluation group (EG) consisted of additional 10 plans, similar to those included in the model libraries. In the comparative study, the plans manually optimized by different planners with Eclipse were compared to the plans automatically optimized with RapidPlan™. RapidPlan™ optimized plans will be defined as KBP plans, while the plans generated with Eclipse without the help of RapidPlan™ are defined as manual plans. Optimization and dose calculation were performed using the progressive resolution optimizer and the anisotropic analytical algorithm with a 2.5mm calculation grid. The plans included in EG were not included in the model libraries. Both IMRT plans were generated with the same prescription dose and beam characteristics (beam isocenter, angles and numbers). During the study, 6 planners (2 beginner planners, 2 junior planners and 2 senior planners) with planning experience of 2–10 years interested in the study were engaged in the IMRT planning. One year planning experience at our institution may be equivalent to several years’ experience at other institutions, because we annually treated more than one thousand breast cancer patients. One beginner planner, junior planner and senior planner have generated approximately 120, 600 and more than 1200 breast cancer IMRT plans, respectively.

### Dose comparison

All plans were evaluated and compared by a physician who specializes in breast cancer RT with more than 20 years work experience for their ability to fulfill the plan acceptance criteria. DVHs were calculated for each plan. For PTV_elective_ and PTV_boost_, the lowest dose received by 98% of the volume (D_98%_) and the highest dose received by 2% of the volume (D_2%_) were compared. The dose conformality of PTVs was evaluated by the conformation number (CN) according to the following equation (1):

1 $$ \mathrm{C}\mathrm{N}=\frac{{{\mathrm{PTV}}^2}_{\mathrm{ref}}}{{\mathrm{V}}_{\mathrm{PTV}}\times {\mathrm{V}}_{\mathrm{ref}}}, $$

where PTV_ref_ represented the volume receiving the prescription dose in PTV, V_PTV_ stood for the volume of the PTV, and V_ref_ was the volume that received the prescribed dose. For OARs, D_mean_ and V_x_ to the heart (x = 5, 20 and 30), ipsilateral lung (x = 5, 20 and 30), contralateral breast and contralateral lung were compared. The plan difference was facilitated by defining the relative OAR dose deduction (δ) according to the following equation (2):

2 $$ \updelta = \frac{{\mathrm{P}}_{\mathrm{manual}}\hbox{-} {\mathrm{P}}_{\mathrm{KBP}}}{{\mathrm{P}}_{\mathrm{manual}}}\times 100, $$

where P refers to the dose (Gy) or volume (%).

### Statistical analysis

Statistical analysis was performed to compare the dosimetric differences between manual and KBP plans. Kolmogorov-Smirnov tests for the normal distribution were run for all relevant metrics of all structures. The paired t-test was run for each relevant metric for each planner. There were 36 tests run in total for PTVs and 60 tests for OARs. In consideration of test multiplicity, a *p*-value < 0.0014 was considered statistically significant for PTVs and <0.00083 for OARs after the Bonferroni correction. All statistical analyses were performed with IBM-SPSS statistics, version 19 (SPSS Inc, Chicago, IL).

## Results

### PTV coverage

**Fig. 1:**
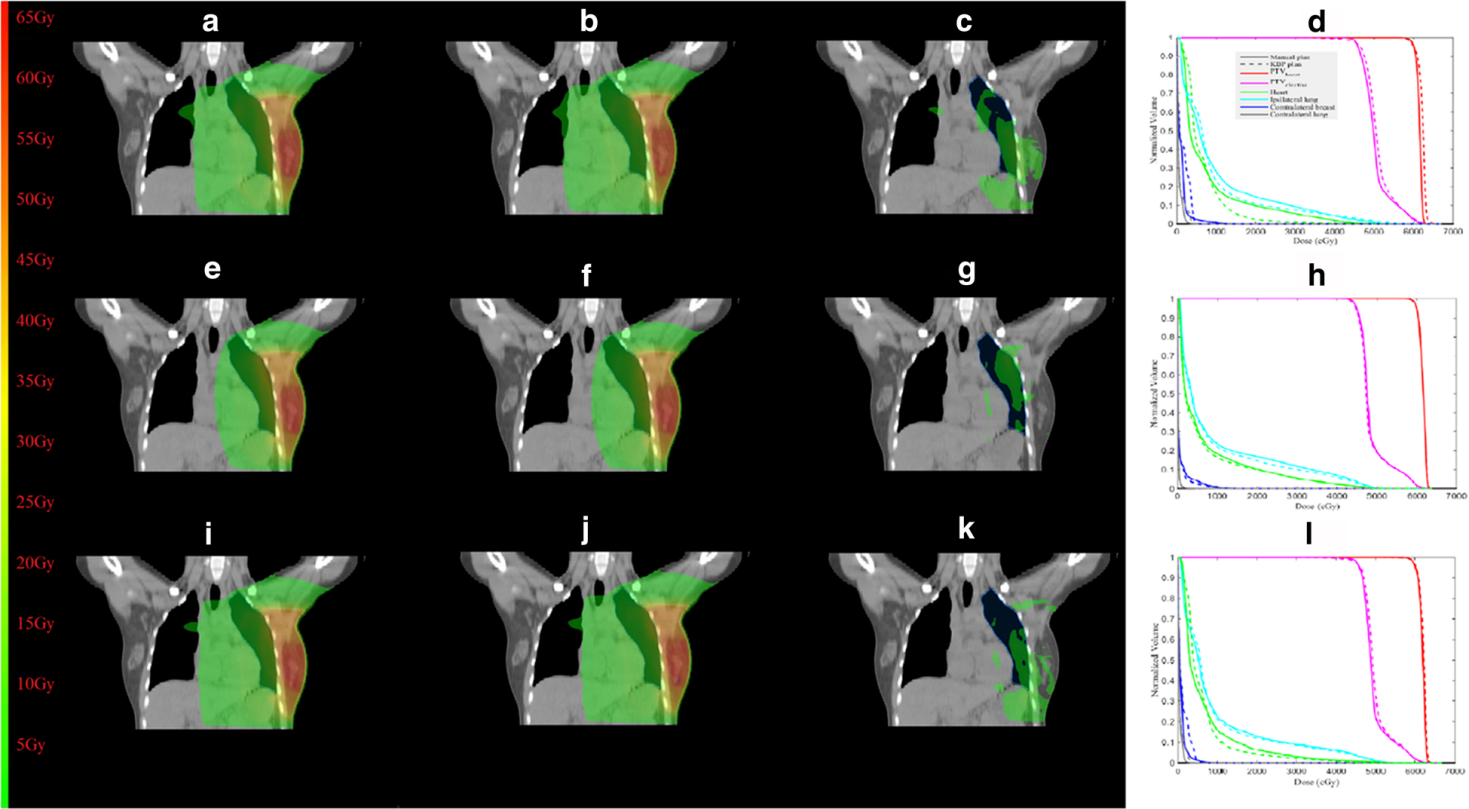
The dose distributions for one representative patient on coronal planes of manual and KBP plans are shown in (**a**) and (**b**) for one beginner planner, (**e**) and (**f**) for one junior planner and (**i**) and (**j**) for one senior planner, respectively. (**c**), (**g**) and (**k**) display the dose differences between (**a**) and (**b**), (**e**) and (**f**) and (**i**) and (**j**), respectively. The dose-volume histograms are shown for (**d**) one beginner planner, (**h**) one junior planner and (**l**) one senior planner

Table 1 presents the detailed statistical analysis of the PTV coverage and CN for the entire EG divided per planner. The dose distributions for one representative patient on coronal planes of manual and KBP plans are shown in Fig. 1(a) and (b) for one beginner planner, Fig. 1(e) and (f) for one junior planner and Fig. 1(i) and (j) for one senior planner, respectively. All plans were capable of achieving the prescription requirement. There were almost no statistically significant differences in terms of the PTV coverage, while the CN was remarkably similar. It clearly showed that the PTV dose distributions were essentially equivalent between the two plans.

**Table 1:** The detailed statistical analysis of the PTV coverage and CN for the entire evaluation group divided per planner

Planner	Plan	PTV_boost_			PTV_elective_		
		D_98%_ (Gy)	D_2%_ (Gy)	CN	D_98%_ (Gy)	D_2%_ (Gy)	CN
Beginner planner A	Manual plan	59.54	63.77	0.70	45.39	57.69	0.65
	KBP plan	59.45	63.95	0.72	45.37	57.86	0.66
	*p* value	0.06	0.20	0.07	0.79	0.39	0.38
Beginner planner B	Manual plan	59.43	63.79	0.73	45.81	57.75	0.65
	KBP plan	59.38	64.16	0.73	45.66	57.35	0.70
	*p* value	0.52	0.13	0.38	0.40	0.33	0.05
Junior planner A	Manual plan	59.35	65.24	0.74	45.15	56.88	0.69
	KBP plan	59.19	65.34	0.73	45.09	56.85	0.68
	*p* value	0.24	0.82	0.14	0.72	0.91	0.20
Junior planner B	Manual plan	59.53	63.63	0.74	44.95	58	0.69
	KBP plan	59.33	63.53	0.73	44.55	58.39	0.69
	*p* value	0.17	0.55	0.44	0.05	0.16	0.88
Senior planner A	Manual plan	59.43	64.57	0.77	45.85	57.4	0.68
	KBP plan	59.44	64.91	0.75	45.91	58.12	0.69
	*p* value	0.86	0.07	0.06	0.76	0.11	0.17
Senior planner B	Manual plan	59.42	64.85	0.72	45.92	57.18	0.70
	KBP plan	59.43	64.06	0.74	46.14	57.94	0.70
	*p* value	0.93	0.08	0.23	0.08	0.05	0.37

Data shown are the averages of the respective parameters for the 10 patients

### OARs sparing

The summary of the quantitative analysis of the OARs sparing for the entire EG is listed in Table 2. Figure 1(d), (h) and (l) show the comparisons of DVHs for one representative patient, respectively. The distributions of all DVH metrics were sufficiently similar to normal distributions. Overall, the results demonstrated that the doses to most of OARs were on average lower or equal in KBP plans compared to manual plans except for the senior planners, where the very small differences were not statistically significant. Notably, KBP data showed a systematic trend to have superior dose sparing at most parameters for the heart and ipsilateral lung without adding doses to the other OARs or compromising the PTV coverage. The improved OARs sparing could be achieved, particularly for the beginner and junior planners. Many differences were statistically significant. Besides, the variability in the doses to all OARs was effectively reduced between KPB plans generated by planners with different planning experiences.

**Table 2:** The summary of the quantitative analysis of the OARs sparing for the entire evaluation group for each planner

Planner	Plan	Heart				Ipsilateral lung				Contralateral breast	Contralateral lung
		V_5_ (%)	V_20_ (%)	V_30_ (%)	Mean dose (Gy)	V_5_ (%)	V_20_ (%)	V_30_ (%)	Mean dose (Gy)	Mean dose (Gy)	Mean dose (Gy)
Beginner planner A	Manual plan	39.80	10.89	5.73	7.50	58.13	18.42	12.09	11.60	0.43	0.20
	KBP plan	36.87	7.49	4.23	6.62	51.56	16.59	11.15	11.43	0.47	0.20
	*p* value	0.002	<0.0001	0.001	<0.0001	<0.0001	0.001	0.01	0.33	0.32	0.95
Beginner planner B	Manual plan	39.37	11.28	6.03	7.52	58.15	18.31	12.12	11.85	0.47	0.20
	KBP plan	37.73	7.80	4.13	6.63	53.46	16.21	10.52	11.47	0.48	0.16
	*p* value	0.30	<0.0001	<0.0001	<0.0001	0.01	0.002	0.004	0.21	0.20	0.06
Junior planner A	Manual plan	36.46	10.25	5.33	6.99	52.3	19.16	12.82	11.70	0.49	0.17
	KBP plan	33.85	9.73	5.30	6.61	50.52	18.88	12.49	11.51	0.43	0.17
	*p* value	<0.0001	0.04	0.87	<0.0001	0.03	0.32	0.44	0.41	0.18	0.17
Junior planner B	Manual plan	35.36	10.41	5.54	7.00	53.75	18.56	11.94	11.78	0.41	0.17
	KBP plan	34.63	9.63	5.20	6.6	51.92	17.98	11.75	11.41	0.41	0.17
	*p* value	0.23	0.02	0.04	<0.0001	0.02	0.10	0.42	0.05	0.88	0.82
Senior planner A	Manual plan	36.58	8.00	4.53	6.72	53.76	18.51	12.76	11.79	0.46	0.22
	KBP plan	37.80	7.76	4.64	6.63	54.42	18.63	12.73	11.94	0.47	0.19
	*p* value	0.06	0.23	0.41	0.14	0.39	0.70	0.91	0.48	0.24	0.27
Senior planner B	Manual plan	36.78	7.98	4.59	6.70	54.07	17.97	12.16	11.74	0.47	0.21
	KBP plan	37.00	7.74	4.58	6.55	54.32	17.8	12.07	11.73	0.47	0.19
	*p* value	0.71	0.40	0.90	0.02	0.77	0.60	0.68	0.98	0.70	0.15

Data shown are the averages of the respective parameters for the 10 patients

**Fig. 2:**
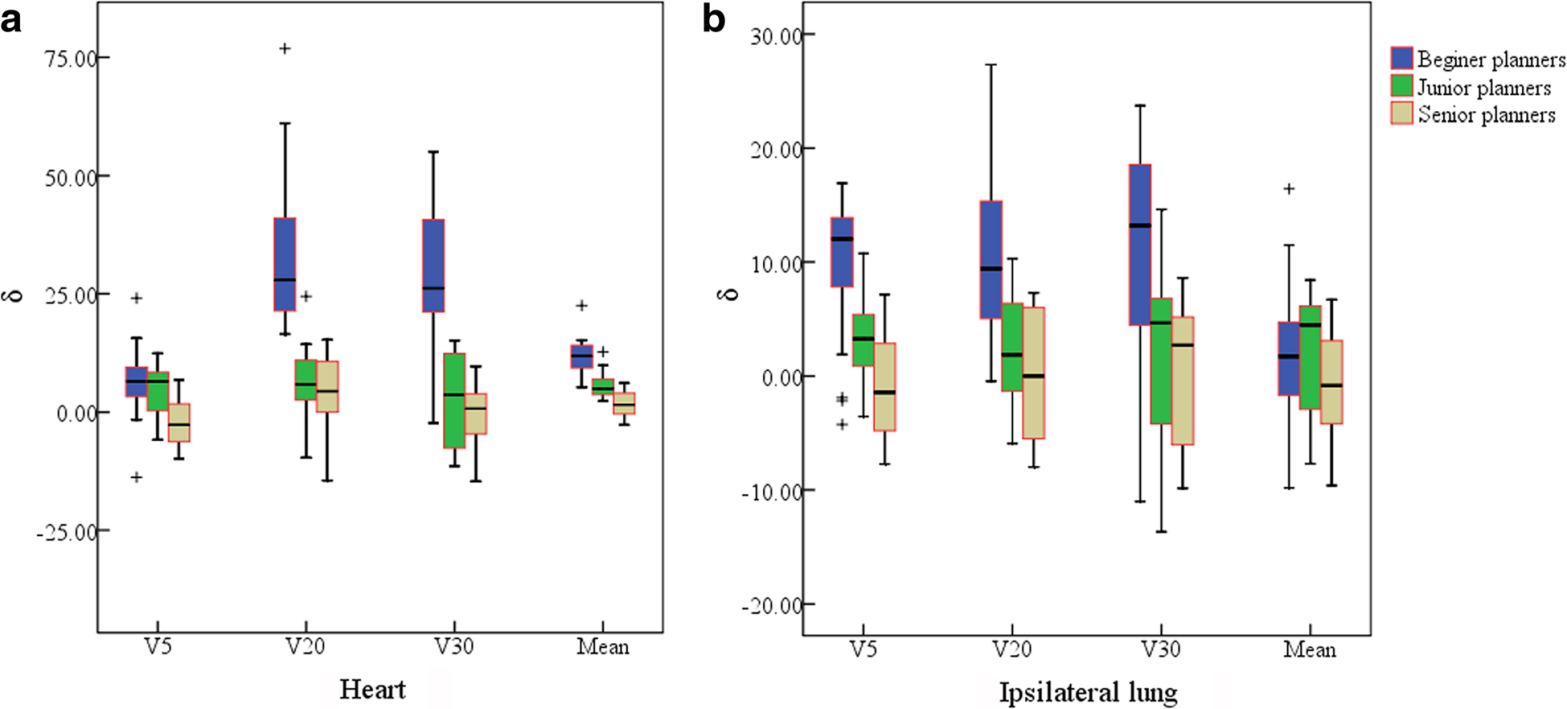
The box plots depicting the effect of KBP implementation on (**a**) the heart and (**b**) the ipsilateral lung dose for planners with each level of planning experiences. δ means the relative OAR dose deduction

Figure 1(c), (g) and (k) display the dose differences between Fig. 1(a), (b), (e), (f), (i) and (j), respectively. The major dose differences are mainly caused by adoption of KBP which led to varying degrees of improved OARs sparing for different planners. Figure 2 shows the effect of KBP implementation on (a) the heart and (b) the ipsilateral lung dose for planners with each level of planning experiences. On average, the heart δ for the beginner planners was 5.1% for V_5_, 32.3% for V_20_, 29.4% for V_30_ and 10.9% for D_mean_, respectively; while for the junior planners was 4.7% for V_5_, 6.46% for V_20_, 2.9% for V_30_ and 5.1% for D_mean_, respectively. For the ipsilateral lung, the average δ for the beginner planners was 9.5% for V_5_, 10.7% for V_20_, 10.4% for V_30_ and 2.0% for D_mean_, respectively; while for the junior planners was 2.9% for V_5_, 2.3% for V_20_, 1.9% for V_30_ and 2% for D_mean_, respectively. For the senior planners, KBP was found to have no noticeable effect on these OARs sparing.

## Discussion

This study demonstrated the feasibility of implementing KBP to assist IMRT planning for left-sided breast cancer patients. This is in line with Fogliata et al., pioneers in implementing KBP in breast cancer, who provided the evidence about the dosimetric quality and effectiveness [19]. Furthermore, we quantified the potential gains of KBP for planners with different planning experiences. KBP proved to be useful in guiding the beginner and junior planners to improve the plan quality without any manual intervention. Based on our results, KBP plans provided at least comparable plan quality compared to manual ones. In addition, adoption of KBP was able to improve the consistency of IMRT plans by minimizing the variations of plan quality due to individual planner differences. KBP showed promise for homogenizing plan quality by transferring planning expertise from more experienced to less experienced planners. Moreover, our results also showed that the experienced planners derived fewer benefits from KBP.

Left-sided breast cancer patients received IMRT with SIB were chosen for the analysis. Besides the prevalence of the cancer, there are multiple PTVs and many individual OARs that need to be balanced. Therefore, we considered it was an appropriate clinical scenario to test the performance of KBP, for relatively demanding plans. The development and implementation of heart-sparing breast RT techniques remains an international priority. Breath-hold techniques [23–28] and VMAT [28] improve the heart sparing. Both techniques might be advisable for breast RT, but they have not yet been widely implemented at our institution due to resource costs and staff training. The study was limited to IMRT delivered to free-breathing patients.

Our data showed that KBP plans were similar to manual ones in the PTV coverage and dose conformality. Most importantly, the doses to most of OARs were on average lower or equal in KBP plans compared to manual ones. The finding of improved OARs sparing could be explained by suboptimal dose–volume constraints being used in the historical manual-planning cohort. Encompassing the breast plus tumor bed without increasing the risks of cardiac complication probabilities is a great technical challenge when the surgical bed location is close to the heart. Fifteen-year cardiac mortality was higher for patients who received RT for left-sided compared to right-sided breast cancer [29]. Patients with early-stage breast cancer have a high probability of long-term survival; parts of the survival gain could be offset by the non-breast related mortality and morbidity from late cardiovascular damage due to the cardiac exposure to radiation in the long time follow-up. Therefore, the critical goal of breast RT should always be to minimize the irradiated heart volume as much as possible (without compromising PTV coverage). Decreases of heart D_mean_ by more than 10% with KBP were found in 25% of the plans. Darby et al. [30] showed a linear increase in the relative rate of major coronary events with the heart D_mean_. 7.4%/Gy is the excess relative risk per Gy. Based on this, a reduction of 0.89Gy for the beginner planners could represent an approximately 6.6% improvement in long-term major coronary events and a reduction of 0.39Gy for the junior planners could represent an approximately 3.0% improvement. It should be stressed, however, that this is highly patient-specific and will be influenced by other factors. More than 45% patients had the relative reduction of heart V_30_ by more than 10% with KBP. Previous studies [31, 32] of early breast cancer and Hodgkin’s disease have shown that the heart V_30_ may also result in few cardiac complications. However, looking over the studies [30, 33] of radiation-related heart disease of breast cancer patients and atomic bomb survivors, there are no known “safe” levels of radiation to the heart. Therefore, the heart should be spared as much as possible. Furthermore, the ipsilateral lung sparing is an additional concern for breast cancer patients. The quantitative analysis of normal tissue effects in the clinic project reviewed several dose–response studies for lung, and found that there were usually strong correlations between various dosimetric parameters (e.g., V_5_ and V_20_) and pneumonitis risk [34]. Our results suggested that KBP plans were superior to manual ones in ipsilateral lung V_20_ especially for the beginner planners.

Various alternative solutions are being proposed to assist planners efficiently guiding clinical tradeoffs among the competing objectives (PTV coverage, OARs sparing and practical feasibility) and decide the clinically optimal balance, such as AutoPlanning [35] and multicriteria optimization [36–42]. AutoPlanning aims to use an iterative algorithm based approach to automatically adapt objectives, constraints and dose shaping structures during the planning optimization process to achieve clinical goals. Multicriteria optimization starts from static clinical objectives and navigates through the realm of possible solutions to find the Pareto optimal plan. Both approaches are time-consuming and also require profound knowledge, experience and interaction to determine the optimal plan, which poses special difficulty of correlating the anatomy and dosimetry relations.

One limitation of the current study is that IMRT plans in the model libraries were generated with Pinnacle^3^ and the new ones with Eclipse. The large difference between the optimization algorithm and dose calculation may affect the estimations and statements about the accuracy of the model libraries. Another limitation is the range of patient geometries in the model libraries, which still may not represent the full diversity of breast cancer cases due to individual differences. Special caution should be taken when applying the model libraries to those patients whose geometry falls outside the range of the constituent plans in the libraries. In addition, the estimated DVH curves imply the information about physician-approved dose-volume constraints and represent acceptable tradeoffs for the specific patient. The accuracy of the estimations mainly depends on the quality of IMRT plans in the model libraries. However, the optimization may be compromised to a certain extent due to the planning complexity and other clinical constraints. Suboptimal plans in the model libraries may degrade results with the KBP approach. Deeper analyses on the goodness of the estimation model configuration in terms of the model size, plan and anatomy homogeneity are required in the future.

## Conclusions

It is feasible to generate acceptable IMRT plans after implementing KBP for left-sided breast cancer. KBP helps to effectively improve the quality of IMRT plans against the benchmark of manual plans for less experienced planners without any manual intervention. KBP showed promise for homogenizing the plan quality by transferring planning expertise from more experienced to less experienced planners. More training and fine-tuning might further improve the performance of KBP, which will be further explored at our institution.

## Abbreviations

CN: The conformation number
D_2%_: The highest dose received by 2% of the volume
D_98%_: The lowest dose received by 98% of the volume
D_mean_: The mean dose
DVH: Dose–volume histogram
EG: Evaluation group
IMRT: Intensity modulated radiotherapy
KBP: Knowledge-based planning
OAR: Organ-at-risk
P: The dose or volume of organ-at-risk
PTV: Planning target volume
PTV_boost_: The boost PTV
PTV_breast_: The breast PTV
PTV_elective_: The elective PTV
PTV_ref_: The volume receiving the prescription dose in the target volume
SIB: Simultaneous integrated boost
TPS: Treatment planning system
VMAT: Volumetric modulated arc therapy
V_PTV_: The volume of the PTV
V_ref_: The volume that received the prescribed dose
V_x_: The percent of volume that at least received xGy
δ: The relative dose deduction
